# Protocol for Autologous Fat Grafting for Immediate Reconstruction of Lumpectomy Defects Following Surgery for Breast Cancer

**DOI:** 10.2196/resprot.5494

**Published:** 2016-07-05

**Authors:** Apresh Singla

**Affiliations:** ^1^ St Vincent's Hopsital Plastic Surgery Sydney Australia

**Keywords:** Fat grafting, lumpectomy defects

## Abstract

**Background:**

For women undergoing breast conservative surgery or lumpectomy for early stage breast carcinoma, there are limited options for reconstruction. Options include the use of flap surgery and/or implants, and have a significant associated morbidity and cost. Autologous fat grafting is a new alternative that can achieve a good cosmetic result, while reducing patient morbidity and cost by avoiding more extensive surgery.

**Objective:**

The primary objectives are to assess patient satisfaction using the Breast-Q questionnaire and to evaluate fat graft volume. The secondary objectives are fat survival and assessment for complication (eg, fat necrosis, cysts), local recurrence, and the number of sessions needed for a satisfactory outcome.

**Methods:**

This study is a case series of 100 patients, at a single-center institute spanning one year. The inclusion criteria include: female sex, age 18 to 75, early state breast cancer (confirmed on ultrasound/ positron emission tomography-computed tomography and cytology), amenable to breast conservative surgery, and at least 6 months post-completion of radiotherapy/ hormone/chemotherapy. Exclusion criteria include patients with more advanced stages of breast cancer necessitating total mastectomy, those unsuitable for surgical excision, and those in whom lumpectomy is not feasible. The patients will have follow-up data collected at 6 months, 12 months and 5 years post-operatively.

**Results:**

This study will begin enrolment in January 2017. We anticipate that there will be good patient satisfaction with fat grafting. The risk for long-term breast cancer recurrence hasn’t been evaluated extensively in literature, however some clinical studies have shown no increased risk of breast cancer in appropriately selected patients at one year. Although some patients may develop complications from fat grafting (eg, necrosis/cysts) this should not confuse the radiological detection of breast cancer recurrence.

**Conclusions:**

Fat grafting is proving to be a viable option for reconstruction of lumpectomy defects with good patient satisfaction. The heterogeneous methods of reporting the harvesting of fat in literature may account for the variable outcomes described, and makes it difficult to compare results with similar studies. The long-term risk of breast cancer recurrence with fat grafting for lumpectomy defects is unknown.

## Introduction

### Disease Background

Women undergoing breast conservative surgery or lumpectomy for early stage breast carcinoma currently have limited options for breast reconstruction. Surgical options include the use of flaps and implants, and have a significant associated morbidity and financial cost.

Autologous fat grafting is a new alternative technique that can achieve a good cosmetic result, while reducing patient morbidity and cost by avoiding more extensive surgery. There is limited literature describing the role of autologous fat grafting for lumpectomy defects. There is, however, a prospective case controlled study (the RESTORE 2 trial) that has demonstrated adequate graft survival, good breast contour, patient satisfaction, and low complication rates [1-3]. Another large retrospective study followed patients who underwent autologous fat grafting, following breast conservative surgery for breast cancer or subsequent breast reconstruction, over a period of 10 years. This study also demonstrated a low complication rate and no long-term increased risk of breast cancer recurrence [4].

### Rationale for Performing the Study

The aim of our study is to establish the feasibility of autologous fat grafting for lumpectomy defects in a large cohort of patients, and to identify factors associated with improved graft survival and better patient satisfaction.

## Methods

### Primary Objectives

We aim to assess patient satisfaction using the autologous fat grafting technique (using the patient-reported Breast-Q questionnaire), and fat graft volume (using radiological techniques).

### Secondary Objectives

We will assess fat survival and complications (eg, fat necrosis, cysts) using clinical and radiological techniques, and local recurrence using oncological methods. The process will be assessed to determine the number of sessions needed for a satisfactory outcome.

### Study Design

**Figure 1:**
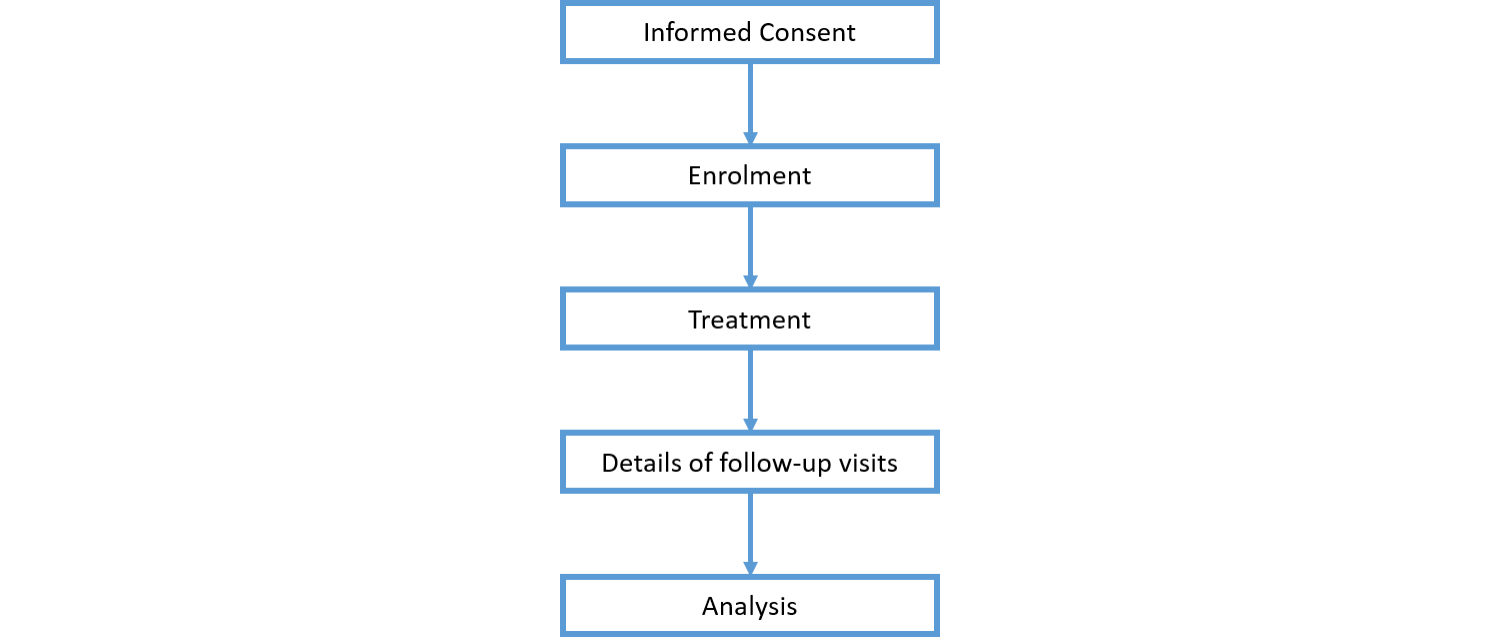
Study flow chart.

This study will be a case series and will aim to recruit 100 patients over a period of one year. All procedures will be performed through St. Vincent’s Public Hospital, and will follow the process outlined in Figure 1. No formal statistical calculations were used to arrive at a sample size of 100.

### Inclusion and Exclusion Criteria

Female patients aged 18-75 who have early stage breast cancer amenable to breast conservative surgery will be included. Confirmation of breast cancer or *in situ* carcinoma must be determined via ultrasound and/or positron emission tomography-computed tomography and cytology, and patients must give written informed consent to participate and comply with follow-up. Patients must also be at least 2 years post-completion of breast conservative surgery and adjuvant radiotherapy, hormone treatments, and/or chemotherapy. Exclusion criteria include patients with more advanced stages of breast cancer necessitating total mastectomy, or those unsuitable for surgical excision. Patients with small breasts, in whom lumpectomy is not feasible, will not be included in the study.

### Investigation Plan

Following consent, baseline characteristics will be recorded in an online database. Details will include age, smoking, co-morbidities, unilateral versus bilateral disease, histopathological factors (location of lesion, benign vs malignant tumor, size of lesion, size of total resection, minimum clearance margin, and time since resection), and any chemotherapy, hormone treatment, or radiotherapy.

The procedure of autologous fat grafting involves several steps. The liposuction site is initially infiltrated with combined saline solution and diluted adrenaline solution, and the adipose tissue is subsequently suctioned using a standard cannula with a conventional liposuction machine. The amount of fat tissue to be grafted is determined by the surgeon at the time of the procedure. The fat graft is subsequently centrifuged to separate out adipose tissue, which is then inserted into the breast. The amount of fat grafted will be recorded in the patient’s notes.

Patients will be followed-up in the outpatient clinic to assess graft survival and potential complications at 6 months, 12 months, 24 months, and 5 years. Follow-up visits will entail a physical examination and an ultrasound. In addition, a physical examination will also be undertaken post-operatively at 1 week to detect early complications. Details for each visit are presented in Table 1.

**Table 1:** The list of interventions at the enrollment visit and at subsequent follow up intervals.

List of Interventions	Enrolment Visit	6 Months	12 Months	5 years
Informed Consent	✓			
Inclusion / exclusion criteria	✓			
Physical examination		✓	✓	✓
Ultrasound		✓	✓	✓
Adverse Event & Serious Adverse Event Assessment		✓	✓	✓

The follow-up protocol for this study differs from the standard of care only in that ultrasound will be used to assess for potential complications relating to fat grafting. The additional costs associated with ultrasounds will be undertaken by the Plastics and Reconstructive Surgery Department. All other aspects of the follow-up (ie, physical examination and adverse event assessment at the specified time periods) are considered standard follow-up for both Plastic and Reconstructive Surgery and Surgical Oncology.

### Study Procedure Risks

Important risks associated with this study relate primarily to the autologous fat graft. At the time of operation there will be discomfort and pain at the donor site. The main complications of the graft include fat necrosis, cyst formation, and calcification. These issues will be monitored via the use of an ultrasound at the specified time periods.

Furthermore, the role of autologous fat grafting in local cancer recurrence in lumpectomy patients is not known, as no long-term trials have been conducted. Earlier studies examining this issue have suggested no increased risk one year after the procedure [1].

### Recruitment and Screening

Following review in the breast cancer multidisciplinary clinic at St. Vincent’s Public Hospital, and having being deemed suitable for this study, patients will be offered autologous fat grafting for repair of their lumpectomy defects.

### Informed Consent Process

After the patient is identified as suitable, informed consent will be obtained to enroll the individual in the study. The patient will be informed about how the procedure is conducted, the expected post-operative recovery time, and the follow-up time periods. The risks and benefits of the procedure will also be explained. Finally, the patient will be made aware of suitable alternatives to autologous fat grafting for repair of lumpectomy defects, which will predominately be conservative treatment [5].

### Enrolment Procedure

Individuals will be enrolled in the study after the informed consent process has been completed, and the participant has satisfied all inclusion and exclusion criteria. Participants will receive study enrolment numbers which will be documented in each participant’s medical record and on all study documents. Patient data will be stored in a de-identified format, and will be kept on a secure server in the Department of Plastics and Reconstructive Surgery.

### Adverse Event Reporting

An adverse event is any untoward medical occurrence that results in the following: death, a life-threatening situation, requirement of inpatient hospitalization or prolongation of existing hospitalization, persistent or significant disability or incapacity, congenital or birth defect, or a condition requiring medical or surgical intervention. An adverse event can therefore be any unfavorable or unintended sign, symptom, or condition, or an observation that may or may not be related to the study treatment.

### Confidentiality, Storage, and Archiving of Study Documents

All participants will be issued a study code and all information will be stored in a de-identified format during the study. Following publication of the results, research data will be non-identifiable, and will be stored in a secure computer server for 15 years in the Department of Plastics and Reconstructive Surgery.

## Results

This study will begin enrolment in January, 2017. We anticipate that there will be good patient satisfaction with fat grafting. The risk for long-term breast cancer recurrence has not been evaluated extensively in the literature, however some clinical studies have shown no increased risk of breast cancer in appropriately selected patients after one year. Although some patients may develop complications from fat grafting (eg, necrosis or cysts), these issues should not confuse the radiological detection of breast cancer recurrence.

## Discussion

Fat grafting is proving to be a viable option for reconstruction of lumpectomy defects, with good patient satisfaction. The heterogeneous methods of reporting the harvesting of fat in the literature may account for the variable outcomes described, and makes it difficult to compare results with similar studies.

The risk of breast cancer recurrence is cited as the major risk factor with fat grafting. Furthermore, there is ongoing disparity between *in vivo* and *in vitro* studies. Currently, clinical studies do not demonstrate any increased risk of recurrence with fat grafting of lumpectomy defects, but recent laboratory studies have suggested that fat-derived stem cells can make human breast cancer cells more aggressive in animal models [6]. This finding highlights the need for ongoing research in this area.
